# A novel robust network construction and analysis workflow for mining infant microbiota relationships

**DOI:** 10.1128/msystems.01570-24

**Published:** 2024-12-31

**Authors:** Wei Jiang, Yue Zhai, Dongbo Chen, Qinghua Yu

**Affiliations:** 1 Laboratory of Microbiology, Immunology, and Metabolism, Diprobio (Shanghai) Co., Limited, Shanghai, China; University of California, San Francisco, California, USA

**Keywords:** infant microbiota, microbial network, co-existence network, network robustness

## Abstract

The gut microbiota plays a crucial role in infant health, with its development during the first 1,000 days influencing health outcomes. Understanding the relationships within the microbiota is essential to linking its maturation process to these outcomes. Several network-based methods have been developed to analyze the developing patterns of infant microbiota, but evaluating the reliability and effectiveness of these approaches remains a challenge. In this study, we created a test data pool using public infant microbiome data sets to assess the performance of four different network-based methods, employing repeated sampling strategies. We found that our proposed Probability-Based Co-Detection Model (PBCDM) demonstrated the best stability and robustness, particularly in network attributes such as node counts, average links per node, and the positive-to-negative link (P/N) ratios. Using the PBCDM, we constructed microbial co-existence networks for infants at various ages, identifying core genera networks through a novel network shearing method. Analysis revealed that core genera were more similar between adjacent age ranges, with increasing competitive relationships among microbiota as the infant microbiome matured. In conclusion, the PBCDM-based networks reflect known features of infant microbiota and offer a promising approach for investigating microbial relationships. This methodology could also be applied to future studies of genomic, metabolic, and proteomic data.

**IMPORTANCE:**

As a research method and strategy, network analysis holds great potential for mining the relationships of bacteria. However, consistency and solid workflows to construct and evaluate the process of network analysis are lacking. Here, we provide a solid workflow to evaluate the performance of different microbial networks, and a novel probability-based co-existence network construction method used to decipher infant microbiota relationships. Besides, a network shearing strategy based on percolation theory is applied to find the core genera and connections in microbial networks at different age ranges. And the PBCDM method and the network shearing workflow hold potential for mining microbiota relationships, even possibly for the future deciphering of genome, metabolite, and protein data.

## INTRODUCTION

### Importance of infant microbiota

The relationship between gut microbiota and human health has become a prominent research topic, with growing evidence linking the microbiota to the host’s gut barrier function, dietary metabolism, immune system, and neurodevelopment (1, 2). Over the past decade, advances in next-generation sequencing technologies, such as 16S rRNA sequencing, metagenomics, and metatranscriptomics, have enabled high-throughput, efficient profiling of the gut microbiome’s structure and function (3). As a subset of microbiota research, the infant gut microbiome has attracted significant attention due to its dynamic maturation process and the many factors that influence its development (4–6). Cohort and longitudinal studies have shown that the infant gut microbiota plays a crucial role in the maturation of the immune system and the development of cognitive function, particularly during the first 3 years of life (7–9). Research from various countries has demonstrated that microbiota diversity in infants continues to increase until around 24 months of age (10, 11). As a result, the key bacteria influencing gut health vary significantly across different stages of infant development (12, 13). For instance, during the first month of life, newborns inherit most of their gut microbiota from their mothers, with *Escherichia coli* being the most influential species (14). Between 1 and 3 months, breastfeeding becomes the primary factor shaping the gut microbiota, with *Bifidobacterium* dominating. From 6 to 12 months, as infants are exposed to more environmental factors, other bacteria begin to colonize the gut. Between 12 and 24 months, dietary changes further influence the gut microbiota composition. According to the hygiene hypothesis, the infant microbiota is influenced by complex factors, including delivery mode, breastfeeding, antibiotic use, and pet ownership (15). While numerous studies, particularly from developed countries, have identified environment-specific bacteria in the infant gut, scientists are increasingly recognizing that the interactions among gut bacteria and their contributions to host health are underexplored. Recently, there has been a shift toward focusing on the relationships between bacteria and core taxa during the maturation process of the infant gut microbiota (16, 17).

### Network analysis on infant microbiota association

Given the variability of infant microbiota, advanced bioinformatic methods are crucial for understanding the relationships between bacteria as infant ages and the microbiota matures (18, 19). Microbial networks, which are a form of exploratory data analysis, can reveal the beneficial or competitive interactions between different bacteria and identify core or keystone species linked to specific health conditions (20, 21). Correlation-based microbial co-occurrence networks are among the most commonly used approaches in this field (22). For example, Liu et al. employed correlation network analysis to identify key taxa, including *Prevotella copri*, *Bacteroides dorei*, and *Bacteroides vulgatus*, in patients with colorectal disease (23). Another widely used network method is Sparse Correlation for Compositional Data (SparCC) (24), which estimates correlations between microbial species by transforming compositional data using log-ratios. Kong et al. (25) used SparCC to construct microbial networks and identified key microbial hubs in patients with autism spectrum disorder who were supplemented with probiotics.

To address the challenges such as data sparsity, compositionality, and noise, new tools for microbial network construction have been developed (26, 27). For instance, CoNet improves the robustness of correlation coefficients through bootstrapping (28), while Sparse InversE Covariance Estimation for Ecological Association Inference uses a regularization strategy to stabilize networks (29). Despite these advancements, challenges remain in the stability and robustness of microbial network methods, due to the complexity of the data and varying technological parameters (8, 30, 31). One major issue is that many networks are based on small data sets, often with fewer than 100 samples (32–35). For example, Zhang et al. conducted a correlation-based network analysis with 11 obese and 12 healthy individuals and found a more complex and interactive network in obesity (35). Many authors have acknowledged that these findings need validation in larger cohorts (25, 27, 28). However, the optimal sample size required to produce stable microbial networks remains unclear. Moreover, similar to the inconsistent relationships observed between bacterial biomarkers and health outcomes, microbial network results can vary between studies. For instance, Miao et al. found that microbial networks had twice the degree of connectivity in healthy individuals compared to those with ulcerative colitis (UC) (34), while a study from the Mediterranean found the opposite—healthy controls had a lower average degree than UC patients (33). Potential confounding factors such as patient demographics, diet, and differences in network methodologies could explain these discrepancies. However, whether networks derived from small sample sizes accurately represent microbial interactions in UC patients—and how much sample size and methodological choices influence these findings—remains uncertain. This highlights the need for a robust, reproducible workflow for microbial network analysis. Additionally, many microbial networks are constructed without thoroughly considering the robustness of the methods used (26). It is crucial to select the appropriate network-building method based on the specific characteristics and heterogeneity of the data (36).

In summary, while current microbial network analyses provide valuable insights and striking visualizations, the use of unreliable methods can lead to inconsistent and non-reproducible findings. A more rigorous approach to constructing and defining microbial networks, along with a robust evaluation strategy, is essential for advancing microbiota research, particularly in the complex and variable infant microbiota.

### Overview of the paper

In this study, we developed the Probability-Based Co-Detection Model (PBCDM), a novel correlation-based approach for constructing microbiota co-existence networks. To assess the impact of sample size on network analysis, we collected publicly available raw 16S amplicon sequencing data of infant stool samples from 34 projects, all of which included metadata, particularly age information. Using sampling without replacement strategy, we then repeatedly sampled microbial data from the collected database, which is processed with a same DADA2 pipeline, with incremental sample size 10, 30, 50, 80, 100, 200, 300, 500, 700, 800, 1,000, and 1,200. We compared the stability and sensitivity of networks derived from these different sample sizes using various network-defining methods, including Spearman correlation, Pearson correlation, SparCC, and our self-defined PBCDM. Additionally, we introduced a network shearing method to optimize complex microbial networks, enabling the identification of keystone taxa and the exploration of bacterial interactions. Finally, we applied this methodology to construct and refine the infant microbiota co-existence network across different age ranges, analyzing the changes in key genera and microbial relationships.

## MATERIALS AND METHODS

### Data pooling and preprocessing

We searched for public infant microbiota studies with 16S rRNA amplicon sequencing data in the European Nucleotide Archive (ENA, https://www.ebi.ac.uk/ena/browser/home) and National Center for Biotechnology Information (NCBI, https://www.ncbi.nlm.nih.gov/Traces/study/). Projects lacking associated sample age information were excluded. A total of 34 project data sets from 23 studies were included, and detailed information about these studies is provided in Table S1. After downloading the sequencing data and metadata, we processed the raw amplicon sequencing data using the DADA2 pipeline (37). Samples with fewer than 20,000 reads for taxonomy were excluded, rarefaction test was performed to validate the depth is enough to profile infant microbiota (Fig. S7). Ultimately, we compiled a data set comprising 17,788 samples from the 34 publicly available projects. The full list of sample sources is shown in Table S1.

Given that the infant microbiota varies significantly across different age ranges, we categorized the samples into eight age ranges: under 1 month (0–1 m), 1–3 months (1–3 m), 3–6 months (3–6 m), 6–12 months (6–12 m), 12–18 months (12–18 m), 18–24 months (18–24 m), 24–36 months (24–36 m), and over 36 months (36m+). The sample sizes for each age group are presented in Table 1.

**TABLE 1:** Sample size and dominant genus at different age ranges

Age ranges	Total sample	Most abundant	Most prevalent
0–1 m	4,003	*Bifidobacterium*	*Streptococcus*
1–3 m	3,264	*Bifidobacterium*	*Bifidobacterium*
3–6 m	2,912	*Bifidobacterium*	*Bifidobacterium*
6–12 m	3,034	*Bifidobacterium*	*Bifidobacterium*
12–18 m	1,401	*Bifidobacterium*	*Bacteroides*
18–24 m	1,333	*Bacteroides*	*Faecalibacterium*
24–36 m	1,112	*Bacteroides*	*[Ruminococcus] torques group*
36m+	729	*Bifidobacterium*	*Collinsella*

### Network definition

#### Pearson correlation network

The Pearson correlation network between genera was constructed for different age groups, using correlation coefficients with a *P* value threshold of <0.05. To satisfy the normality assumption required for the Pearson correlation test, microbiota data from each sample were normalized using the centered log-ratio (clr) transformation (Fig. S8).

#### Spearman correlation network

The Spearman correlation network between genera across different age ranges was constructed using correlation coefficients with a *P* value threshold of <0.05.

#### SparCC network

SparCC was executed with default parameters and 100 bootstrap iterations. Pseudo *P* values were calculated as the proportion of bootstrapped data sets with correlations as extreme as or more extreme than those observed in the original data set. Only correlations with a *P* value <0.05 were retained.

#### Co-existence network with probability-based co-detection model

We constructed a co-existence network based on the co-detection of pairwise genera. The connection in the network was defined as follows:

1. In the selected data set (S), the sample size of S is $n_{s}$; remove the genera that have less than 10% of prevalence.
2. Calculate the prevalence of each genus $p_{i}$ without considering the abundance of a genus.
3. Assume the independence of each genus; for each pair of genera (i, j), calculate the expected sample occurrence in the data set with the prevalence calculated in step 2.
$$Exp_{i,j}=n_{s}*p_{i}*p_{j}$$
4. Calculate the difference (Dev) of expected occurrence (Exp) and observed occurrence (Obs) of genus pair (i, j), and stat the mean and standard deviation of the difference.
$$Dev_{i,j}=Exp_{i,j}-Obs_{i,j}$$
$$M_{Dev}=mean\left(Dev\right)$$
$$\;\;SD_{Dev}=sd\left(Dev\right)$$
5. We observed that biases occurred more frequently within one standard deviation of the mean difference (Fig. S1). This was particularly evident in the range of the mean value minus one standard deviation, suggesting underrepresented observed occurrences. These biases could potentially be attributed to confounding factors such as sequencing region or sample preparation. To address this, we considered differences within one standard deviation as noise and defined the connection between genus *i* and genus *j* as follows:
$$Dev_{i,j}\leq M_{Dev}-1*SD_{Dev},\text{Positive}$$
$$Dev_{i,j}\geq M_{Dev}+1*SD_{Dev},\text{Negative}$$
6. Construct the co-existence network with the qualified connections; the values of $Dev_{i,j}$ will be the intensity of the connections (or edge width).

### Network attributes

#### Closeness centrality

Closeness centrality (*clo*) is a measure of the average shortest distance from each vertex to each other vertex (*v*). Specifically, it is the inverse of the average shortest distance between the vertex and all other vertices in the network (38, 39). The formula is 1 / (average distance to all other vertices).

For a network, the centralized closeness centrality (*Clo*) is defined (40) as follows:


$$Clo=\sum_{v}\left(\max\left(clo_{v}\right)-clo_{v}\right)$$

We use centralized closeness to measure the closeness of the network with R package *igraph* (41).

#### Betweenness centrality

The betweenness centrality (*bet*) captures how much a given node is in between others. This metric is measured with the number of shortest paths (between any couple of nodes in the graphs) that pass through the target node. This score is moderated by the total number of shortest paths existing between any couple of nodes of the graph. The target node will have a high betweenness centrality if it appears in many shortest paths (38, 39).

For a network, the centralized betweenness centrality (*Bet*) is defined (40) as follows:


$$Bet=\;\sum_{v}\left(\max\left(bet_{v}\right)-bet_{v}\right)$$

We use centralized betweenness to measure the betweenness of the network with R package *igraph* (41).

#### Eigenvector centrality

In graph theory, eigenvector centrality (*eig*) is a measure of the influence of a node in a network. It assigns relative scores to all nodes in the network based on the concept that connections to high-scoring nodes contribute more to the score of the node than equal connections to low-scoring nodes (38, 39).

For a network, the centralized eigenvector centrality (*Eig*) is defined (40) as follows:


$$Eig=\;\sum_{v}\left(\max\left(eig_{v}\right)-eig_{v}\right)$$

We use eigenvector centrality to measure the eigenvector centrality of the network with R package *igraph* (41).

### Sample size on network stability

For microbiota profiling data across different age ranges, we performed sampling without replacement at incremental sample sizes of *n* = 10, 30, 50, 80, 100, 200, 300, 500, 700, 800, 1,000, and 1,200, with a maximum of 20 sampling iterations per size. For each sampled data set, we applied four network construction methods and evaluated the resulting networks based on the number of nodes, the number of links per node, and the P/N ratios.

### Co-existence network shearing

After constructing a co-existence network using complete data for a specific age range, network cutting is performed (42). Here, we performed a novel network shearing process to identify the core structure of the network and determine the optimal shearing stop point, drawing parallels to percolation theory in physics (43). The shearing process is as follows:

1. Construct a co-existence network with the edge size as described in the co-existence network definition.
2. Remove the edge with the least edge size regardless of connection type (positive or negative), and calculate the network eigenvector centrality.
3. Remove the edge with the biggest edge size regardless of connection type (positive or negative), and calculate the network eigenvector centrality.
4. If the centrality in step 2 is bigger than 3, the edge with the least edge size is removed; if not, the edge with the biggest edge size is removed.
5. Running steps 2, 3, and 4 until there is only one edge left.

Following the shearing process, we evaluated the trade-off between betweenness centrality and closeness centrality at each shearing step to determine the optimal stopping point. The final core co-existence network was then constructed based on the selected stopping step.

### Statistical analysis

Statistical analysis was conducted using R version 4.3.1. Principal coordinate analysis was performed using Bray–Curtis distance on relative abundance data. Differences in microbiota structure between groups were tested using permutational multivariate analysis of variance. The Shapiro–Wilk test was applied to assess the normality of metrics, including genus abundance, network node numbers, links per node, and positive-to-negative (P/N) ratios. Differences in the number of nodes, links per node, and P/N ratios across different sampling sizes were tested using Student’s *t*-test.

## RESULTS

### The effect of sample size on microbiota network

**Fig 1:**
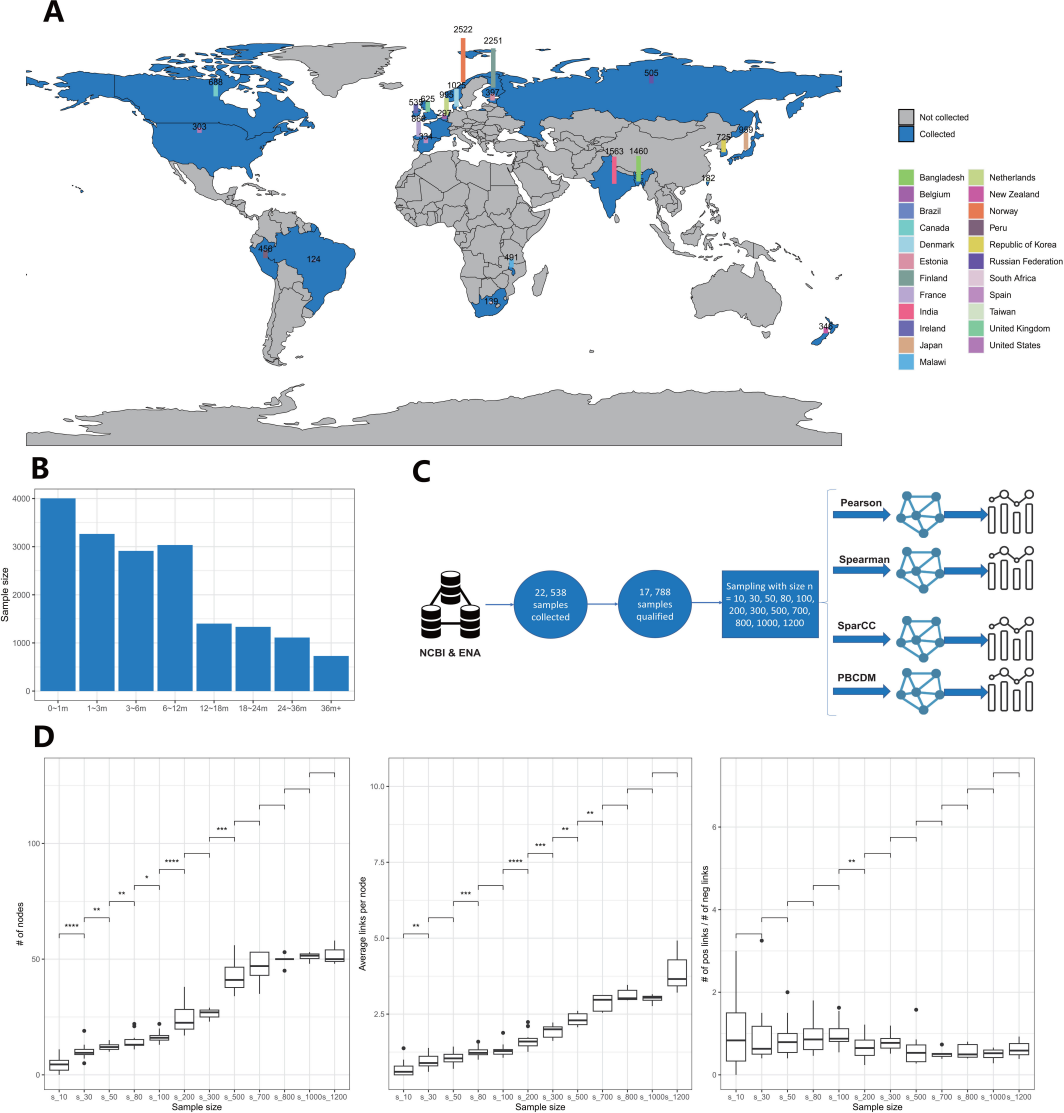
(**A**) The geography distribution of collected qualified samples. (**B**) Sample size per age range. (**C**) Workflow of network stability evaluation. (**D**) The change of network node numbers, average links, and P/N ratios on different sample sizes for SparCC with samples aged 0–1 month.

We collected amplicon sequencing data from infant fecal samples across 34 public projects in 23 countries or regions, available through ENA and NCBI (Fig. 1A). After quality filtering, 17,888 samples were retained for further analysis. The geographical distribution is in Fig. S6, with the majority originating from Europe and Asia (Fig. S6). To account for the influence of infant age on gut microbiota, we categorized the samples into distinct age ranges: 0–1 month (0–1 m), 1–3 months (1–3 m), 3–6 months (3–6 m), 6–12 months (6–12 m), 12–18 months (12–18 m), 18–24 months (18–24 m), 24–36 months (24–36 m), and 36 months+ (36m+) (Fig. 1B). The sample count for each age group is listed in Table 1. For each age group, we obtained over 1,000 samples, except for the 36m+ group, which represents one of the largest data sets in infant microbiota studies. After sequencing data processing, a total of 1,284 genera were detected.

We explored the impact of confounding factors, including source country, delivery mode, breastfeeding pattern, and term mode, on infant microbiota development across different growth stages (Fig. S9 to S12). We then identified the most abundant (highest mean abundance) genera and most prevalent (most frequently detected) genera. *Bifidobacterium* was found to be the most abundant genus in infant microbiota, particularly during the first year of life. For prevalence, *Bifidobacterium* was the most commonly detected genus throughout the first year, although *Streptococcus* was the most prevalent genus in the first month after birth. After the first year, other genera such as *Bacteroides*, *Faecalibacterium*, and *Collinsella* became more frequently detected, reflecting the dynamic and maturing nature of the infant microbiota.

We assessed the stability of the network by testing differences of network attributes on incremental sampling sizes across four different network construction methods: Pearson correlation, Spearman correlation, SparCC, and our self-developed PBCDM (Fig. 1C). We compared network attributes, including node numbers, average links, and P/N ratios, across a range of sample sizes. In most cases, node numbers and average links increased as sample size grew, suggesting that larger data sets detect more correlations. However, the P/N ratio remained relatively stable (Fig. 1D). The full network behavior with varying sample sizes is shown in Fig. S2 to S5 (A–H). Next, we determined the minimum sample size at which these network attributes stabilized—defined as the point where increasing the sample size further showed no significant differences in node numbers, average links, or P/N ratios (Tables 2 to 4). For node numbers, SparCC required at least 200 samples across different age ranges, more than the other methods. In contrast, the Pearson, Spearman, and PBCDM methods reached stable node numbers with fewer than 100 samples. Regarding the average links in the network, correlation methods (Pearson, Spearman) required the most samples to stabilize, indicating that these methods are highly sensitive to sample size. Interestingly, no minimum sample size was observed for age groups like 6–12 m, 18–24 m, and 24–36 m, meaning that even a sample size of 1,200 is insufficient to achieve a stable correlation network for these groups. This suggests that the network’s connections continue to improve with additional samples. A similar phenomenon was observed for SparCC, but the PBCDM-based co-existence network reached stability with the fewest samples, stabilizing at a maximum of 200 samples. For the P/N ratio, the PBCDM method required at most 200 samples to achieve stability, except for the 6–12 m age group. SparCC showed the least sensitivity to sample size, while Pearson and Spearman required the largest sample sizes to stabilize.

**TABLE 2:** Minimum samples required for stable node numbers of different network construction methods

Methods	Pearson	Spearman	SparCC	Co-existence
0–1 m	30	30	500	50
1–3 m	50	80	200	80
3–6 m	80	80	500	100
6–12 m	80	30	700	80
12–18 m	30	50	300	200
18–24 m	30	80	300	30
24–36 m	30	30	300	30
36m+	30	30	200	30

**TABLE 3:** Minimum samples required for stable average network links of different network construction methods

Methods	Pearson	Spearman	SparCC	Co-existence
0–1 m	700	700	700	30
1–3 m	700	700	500	80
3–6 m	800	1,000	700	100
6–12 m	NA	700	500	200
12–18 m	300	500	NA	200
18–24 m	300	NA	300	200
24–36 m	NA	NA	300	50
36m+	200	200	200	80

^*a*^ NA indicates no minimum sample size is detected to stabilize average links.

**TABLE 4:** Minimum samples required for stable P/N ratios of different network construction methods

Methods	Pearson	Spearman	SparCC	Co-existence
0–1 m	300	30	200	30
1–3 m	300	500	50	200
3–6 m	200	200	10	200
6–12 m	NA	200	10	700
12–18 m	200	500	80	200
18–24 m	200	200	80	30
24–36 m	200	80	50	80
36m+	200	200	10	30

^*a*^ NA indicates no minimum sample size is detected to stabilize P/N ratios.

In summary, we concluded that Pearson and Spearman correlation networks were less stable in terms of average links and required larger sample sizes to achieve stable P/N ratios. SparCC required a larger data set to stabilize node numbers and average links, indicating that bigger data sets lead to more complex networks. In contrast, our PBCDM-based co-existence network demonstrated superior stability and robustness, requiring fewer samples to reach stability compared to the other methods.

### Co-existence networks of different age ranges

**Fig 2:**
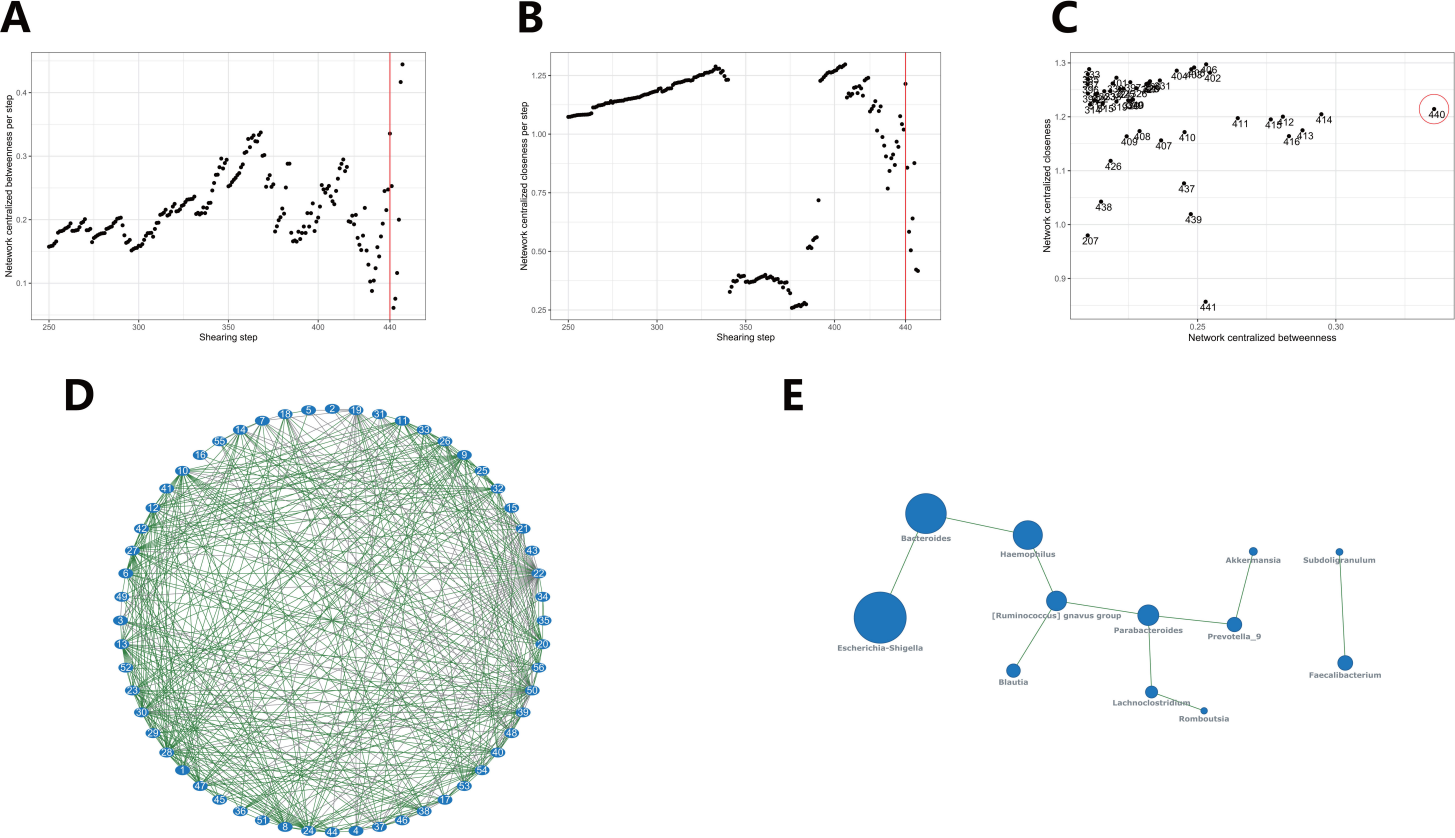
The processing and result of network shearing strategy of infant microbiota aged 0–1 m. (**A**) The change of network centralized betweenness per cutting step, the red line indicates the stopping cutting point. (**B**) The change of network centralized closeness per cutting step, the red line indicates the stopping cutting point. (**C**) The trade-off between network centralized betweenness and closeness, the red circle indicates the stopping cutting point. (**D**) The complex co-existence network before network shearing. (**E**) The final core genera network after network shearing, the node size display the prevalence of the genera in the data set. The edge color indicates the correlation type of two genera, with green as a positive connection and gray as a negative connection.

As demonstrated, our PBCDM network proved to be the most stable and least sensitive to sample size. Using this PBCDM-based method, we constructed a complex microbiota co-existence network for infants across different age ranges, incorporating all available samples from Table 1. Genera detected in fewer than 10% of the samples were excluded. To identify the core genera within the network, we employed a network shearing strategy, progressively removing edges from the initial network to find the optimal structure where network betweenness and closeness are best balanced. For example, for samples aged 0–1 month (Fig. 2), cutting at step 440 provided the best trade-off between network betweenness and closeness. Therefore, we chose to stop the network shearing process at step 440 and defined the network at this point as the core microbiota network (Fig. 2A through C). After the shearing process, the complex co-existence network between genera (Fig. 2D) was denoised, and the core microbial network for infants aged 0–1 month was identified (Fig. 2E).

**Fig 3:**
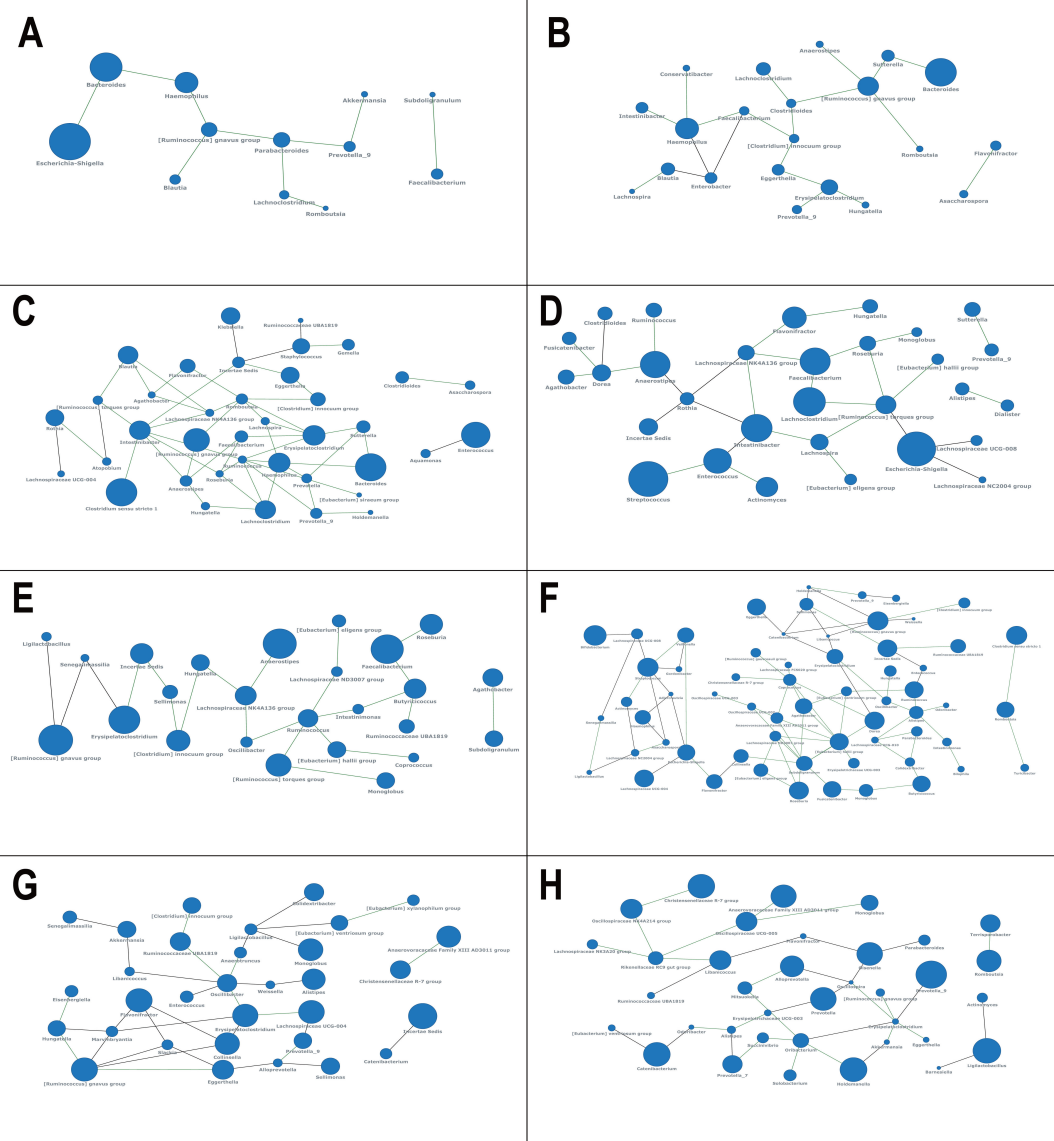
Core co-existence networks of infant microbiota at different age ranges after network shearing. The node size displays the prevalence of the genera in the data set, respectively. The edge color indicates the correlation type of the two genera, with green as a positive connection and gray as a negative connection. (**A**) 0–1 m. (**B**) 1–3 m. (**C**) 3–6 m. (**D**) 6–12 m. (**E**) 12–18 m. (**F**) 18–24 m. (**G**) 24–36 m. (**H**) 36m+.

Using the shearing strategy, we successfully constructed the core co-existence network of infant microbiota across different age ranges (Fig. 3). The complexity of the network increases as the infant grows, reflecting the rising biodiversity and maturation of the microbiota from birth to 3 years old. At 0–1 month, despite the high abundance of *Escherichia* and *Bacteroides*, in the aspect of node eigenvector centrality, the most pivotal genera in the network are *Parabacteroides* and *[Ruminococcus] gnavus group*. From 1 to 3 months, the *[Clostridium] innocuum group*, *Faecalibacterium*, and *Clostridioides* emerge as key genera in the co-existence network, highlighting their importance for newborns. At 3–6 months, 6–12 months, and 12–18 months, as the network becomes more intricate, the shared key genera include *Intestinibacter*, *Ruminococcus*, *Lachnospiraceae NK4A136 group*, *[Ruminococcus] torques group*, and *Anaerostipes*. After 18 months, *Subdoligranulum*, *Collinsella*, and *Oscillibacter* dominate the network, respectively. The key genera contributing most significantly to the networks are listed in Table S2.

### Core co-existence network analysis

**Fig 4:**
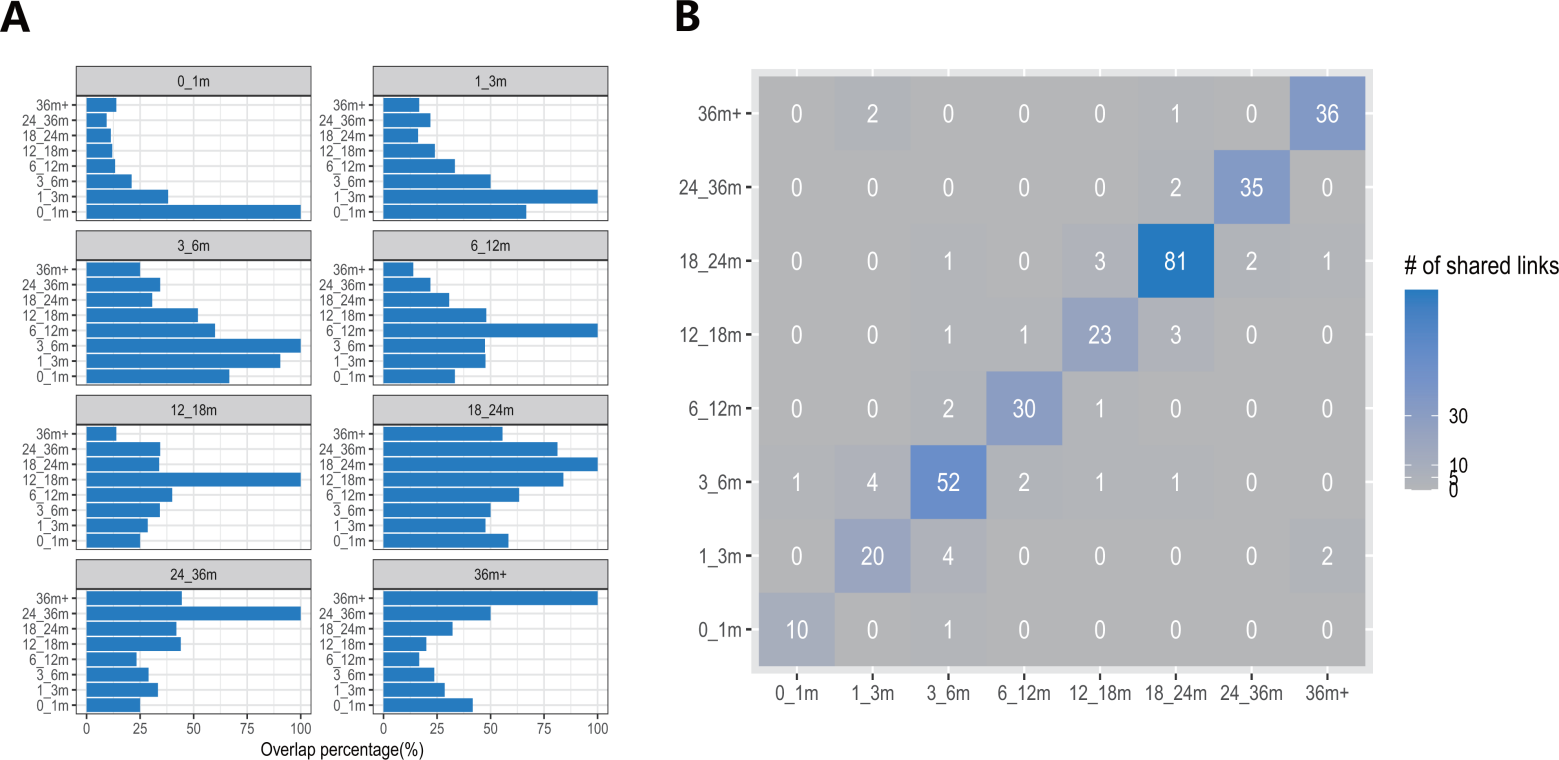
The statistic of core genera and core relationships overlapping between different age ranges. (**A**) Bar plots of core genera similarity across different age ranges, each bar displays the percentage of common genera in other age ranges with the control group. (**B**) Overlapping matrix for connections at different age ranges.

Next, we analyzed the changes in core genera as the infant microbiota matures (Fig. 4). It is evident that in the first month after birth, the core co-existence network is relatively simple, consisting of only 12 genera. The number of core genera increases progressively, reaching a more stable state at 18–24 m, which coincides with the reported saturation of microbiota alpha diversity at this age (6). The common core genera between adjacent age ranges exhibit more overlapping nodes (Fig. 4A), indicating the consistency of the microbiota maturation process. However, we observed very few shared genus-to-genus connections across different co-existence networks, with a maximum of four connections between 1–3 m and 3–6 m age ranges (Fig. 4B). This suggests the ongoing and dynamic nature of infant microbiota development. These results validate the consistency of the microbiota maturation process and, to some extent, demonstrate that the PBCDM-based model is an effective tool for uncovering the patterns in infant microbiota development.

**Fig 5:**
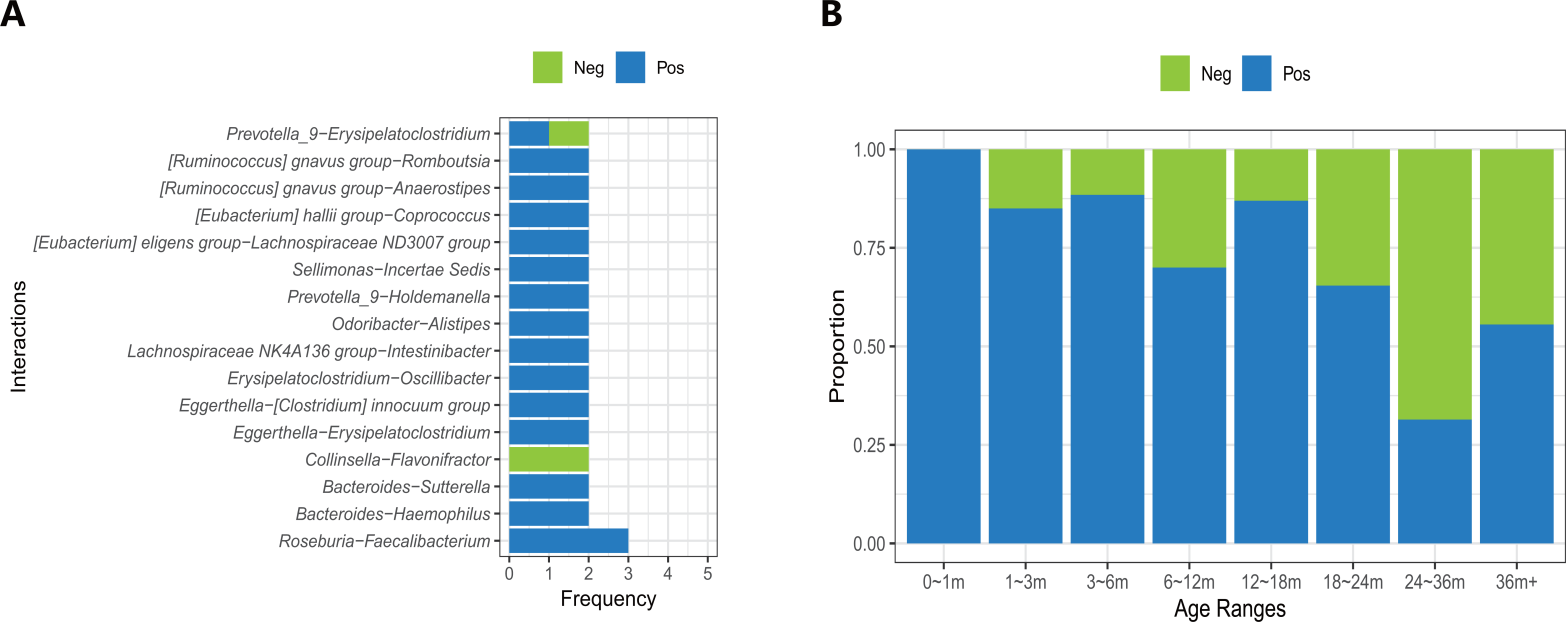
Analysis of core network connections. (**A**) The frequency of overlapped genus-genus connections in the core co-existence networks. (**B**) The proportion of connection types per age range.

We also analyzed the distribution of network links that were detected more than once (Fig. 5A). Most of the common connections are positive, with the exception of a negative correlation observed between *Collinsella* and *Flavonifractor* at 18–24 m and 24–36 m. Notably, *Faecalibacterium* and *Roseburia* emerge as the most frequent co-existing genera with positive correlations in the infant microbiota, appearing consistently at 3–6 m, 6–12 m, and 12–18 m. However, there is an inconsistency in the connection types for *Prevotella 9* and *Erysipelatoclostridium*. These two genera show a positive correlation at 1–3 m and a negative correlation at 36m+, suggesting a shift in the gut environment as the microbiota matures, with distinct ecological dynamics in newborns compared to older children (3+ years old).

We analyzed the proportions of positive and negative connections in the core co-existence network across different age ranges (Fig. 5B). In the 0–1 m age group, no negative connections were observed, suggesting a beneficial microbiota relationship inherited from the mother. However, as the infant matures and experiences increased environmental exposure, the number of negative interactions within the co-existence network increases. Notably, the 24–36 m group exhibited the highest proportion of negative connections, indicating the most complex competitive dynamics within the gut microbiota at this age.

## DISCUSSION

Early-life microbiota has been shown to play a crucial role in long-term health. To better understand the relationship between bacterial communities and health outcomes, a key challenge is to decipher the interactions and core taxa within the infant microbiota, particularly during different developmental stages (44). In this study, we apply network analysis to investigate bacterial interactions in infants using data from public databases. After comparing four different network-defining methods, our results demonstrate that the PBCDM method offers the best stability and robustness, underscoring its potential for microbiota network analysis. Furthermore, we utilized PBCDM and a network-shearing strategy to construct and analyze the core co-existence networks from the collected data. Our findings reveal that the core networks exhibit consistency in their constituents and fewer discrepancies in genus relationships as the infant microbiota matures. These results highlight the effectiveness and robustness of our PBCDM method in microbial network and bacterial interaction analysis.

Network-based analysis has been widely studied and compared in microbiota research (26, 27, 33, 34). While these analyses offer valuable insights into microbial interactions, challenges inherent in microbiome data, such as composition bias, overdispersion, and the sample-to-feature ratio, remain significant obstacles (19). Moreover, a solid workflow for comparing key network behaviors, such as stability and robustness, is still lacking. In many previous studies, network connections were defined using correlation methods like Pearson or Spearman (20). However, our results show that these approaches are highly sensitive to sample size, often leading to unstable network structures. This sensitivity makes them unsuitable for analyzing small microbial data sets and may even introduce false positives in microbial analysis (20). Additionally, we found that SparCC is the most unstable method in terms of network nodes and edges. This instability suggests that as sample size increases, the network structure becomes more complex, which we believe contributes to the inconsistencies observed in previous network analysis conclusions (33, 34). In contrast, we developed a co-existence network that focuses on the probability of bacterial co-detection, which surprisingly demonstrated greater stability and robustness. One possible explanation for this is that stool sample sequencing provides only a snapshot of the microbiota, which is dynamic and continuously changing. As a result, sequencing may overrepresent or underrepresent certain bacteria, making our PBCDM-based co-existence network, which does not rely on bacterial abundance, more stable and robust.

The infant microbiota undergoes a continuous process of change and maturation. In our analysis of the core co-existence networks across different age ranges, we observed increasingly complex core genera structures as the infant grows, aligning with the natural progression of microbiota maturation (7–9). Additionally, our findings revealed greater core genera similarity between adjacent age ranges, reinforcing the validity of co-existence networks in studying infant microbiota relationships. Consistent with previous studies that report a relatively simpler gut microbiota in newborns (45), our results show a straightforward network structure for the 0–1 month age group, with no negative links observed in the core co-existence network. As infants grow, we identified more complex network structures and an increase in negative links, indicative of competitive relationships among bacteria. These changes may be driven by factors such as increased environmental exposure, dietary shifts, and limited or competitive nutrient availability in the gut. Interestingly, network links rarely overlapped across age ranges, with only one genus pair appearing three times: the two butyrate-producing bacteria *Roseburia* and *Faecalibacterium*. Their positive connection has been previously documented (46). The only overlapping negative connection was between *Collinsella* and *Flavonifractor*, consistent with prior studies that highlight the beneficial role of *Collinsella* and the dysbiosis-indicating role of *Flavonifractor* (47, 48). Notably, there was minimal contradiction in link types (positive or negative) for the same genus pairs across networks, further supporting the stability and robustness of our co-existence networks.

However, we identified one notable exception: *Prevotella 9* and *Erysipelatoclostridium* exhibited different relationships between the 1–3 m and 36m+ age groups. In newborn babies, the microbiota’s simplicity and abundance of nutritional resources likely provide a niche large enough to support beneficial interactions between these genera. By 3 years of age, increased environmental contact and dietary diversity may alter the gut environment, transforming their relationship into a competitive one. This hypothesis aligns with findings from a previous study on brain tumor patients, which reported significantly higher levels of microbial extracellular vesicles from *Erysipelatoclostridium* and lower levels from *Prevotella 9*, indicating a competitive dynamic between these genera (49). Further wet-lab experiments are needed to validate and elucidate these findings.

Although our PBCDM method has proven effective in uncovering infant microbiota relationships, there are several limitations to this study. First, most of the sample data we analyzed were sourced from Europe and Asia, potentially introducing bias due to variations in baby-raising practices across different regions. While the 17,888 pooled samples we used provide a robust reference point, particularly given the simpler structure of infant microbiota (45), a larger and more geographically diverse global microbiota database is essential for a comprehensive understanding of infant microbiota relationships. Second, our analysis relied on public 16S rRNA sequencing data to assess network stability and sensitivity. However, this sequencing approach and the collected data limited microbiota profiling to the genus level. To uncover true bacterial relationships, higher taxonomic resolution at the species or strain level is necessary. Furthermore, integrating microbial functions, metabolites, and metatranscriptomics into the analysis would be highly beneficial, as functional and metabolic profiling is critical to understanding bacterial interactions at a deeper level. Third, different species within the same genus can exhibit distinct functions, which may complicate their interactions with other genera or species. This highlights the need for species- and strain-level analyses to refine our understanding of these relationships. Additionally, our study focused on pairwise relationships between genera, but there may also be unidirectional or asymmetrical interactions that were not captured. Developing new methods to identify and analyze these one-way interactions will be an important avenue for future research. Lastly, gut microbiota and bacterial interactions are influenced by numerous factors, such as mode of delivery. For instance, it remains unclear whether bacterial interaction networks differ between infants born via C-section and those born naturally at the same age. Addressing this scaling complexity will require more stringent sampling and higher-quality data. Despite these limitations, our study provides a validated workflow for evaluating network methods, offering a robust framework for researchers to conduct solid microbiota network analyses at the species or strain level in future studies.

## CONCLUSION

In this study, we utilized publicly available infant microbiota data to assess the impact of sample size on microbial network analysis. Our findings revealed that the self-defined PBCDM-based network demonstrated the most stable and robust performance. Using PBCDM, we constructed and optimized infant microbiota co-existence networks through a novel network shearing method. This approach enabled us to analyze the core network, identifying key genera and connections, validating the maturation process of the infant microbiota, and highlighting consistent bacterial interactions as biodiversity increases.

## Data Availability

The public data used in this study can be found in Table S1 with project accession IDs.

## SUPPLEMENTAL MATERIAL

Table S1 msystems.01570-24-s0001.csv Sources of public data used in the paper.

Table S2 msystems.01570-24-s0002.csv Key taxa in core networks of different age ranges.

Supplemental figures msystems.01570-24-s0003.pdf Figures S1 to S12.

Supplemental Table Captions msystems.01570-24-s0004.docx Captions for Tables S1 and S2.

ASM does not own the copyrights to Supplemental Material that may be linked to, or accessed through, an article. The authors have granted ASM a non-exclusive, world-wide license to publish the Supplemental Material files. Please contact the corresponding author directly for reuse.
