# Supplementary material for: A novel robust network construction and analysis workflow for mining infant microbiota relationships
Source: mSystems. 2024 Dec 31;10(2):e01570-24. doi: 10.1128/msystems.01570-24 (PMC11834438; doi:10.1128/msystems.01570-24)
Supplement: Supplemental figures — Figures S1 to S12. [file msystems.01570-24-s0003.pdf]

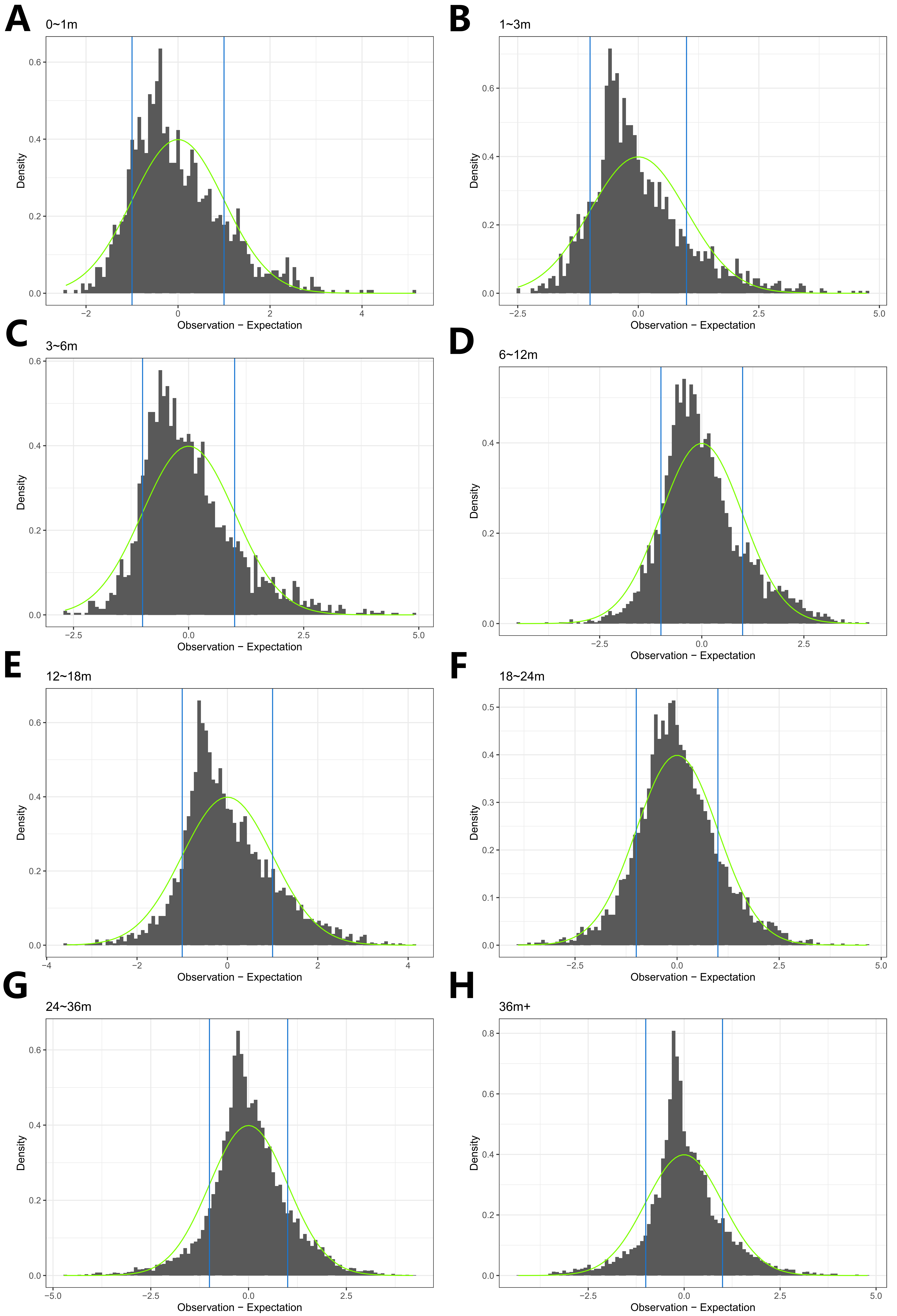

Supplementary Figure 1. The distribution of difference of observed occurrence and expected occurrence at different age ranges. A, 0~1m. B, 1~3m. C, 3~6m. D, 6~12m. E, 12~18m. F, 18~24m. G, 24~36m. H, 36m+. The green curve is the gaussian fitting of the histogram. The blue lines are the boundaries of one standard deviations of the mean.

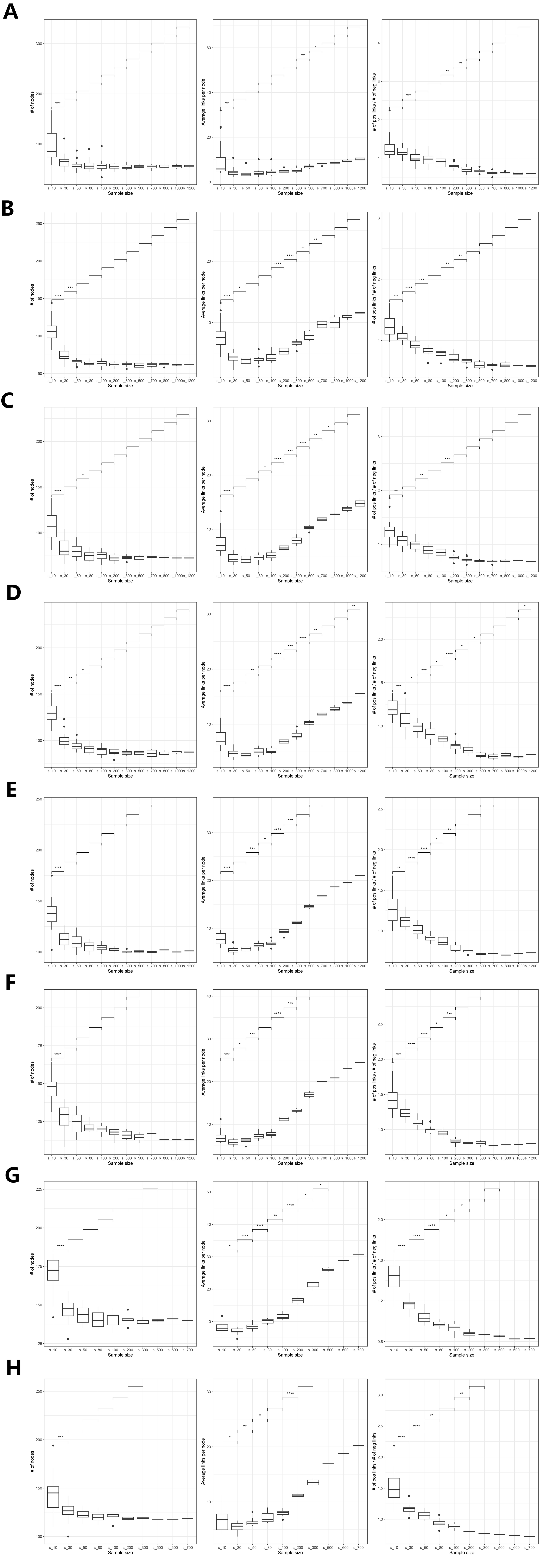

Supplementary Figure 2. The change of network node numbers, average links and positive-to-negative link ratios on different sample sizes for Pearson correlation networks. A, 0~1m. B, 1~3m. C, 3~6m. D, 6~12m. E, 12~18m. F, 18~24m. G, 24~36m. H, 36m+.

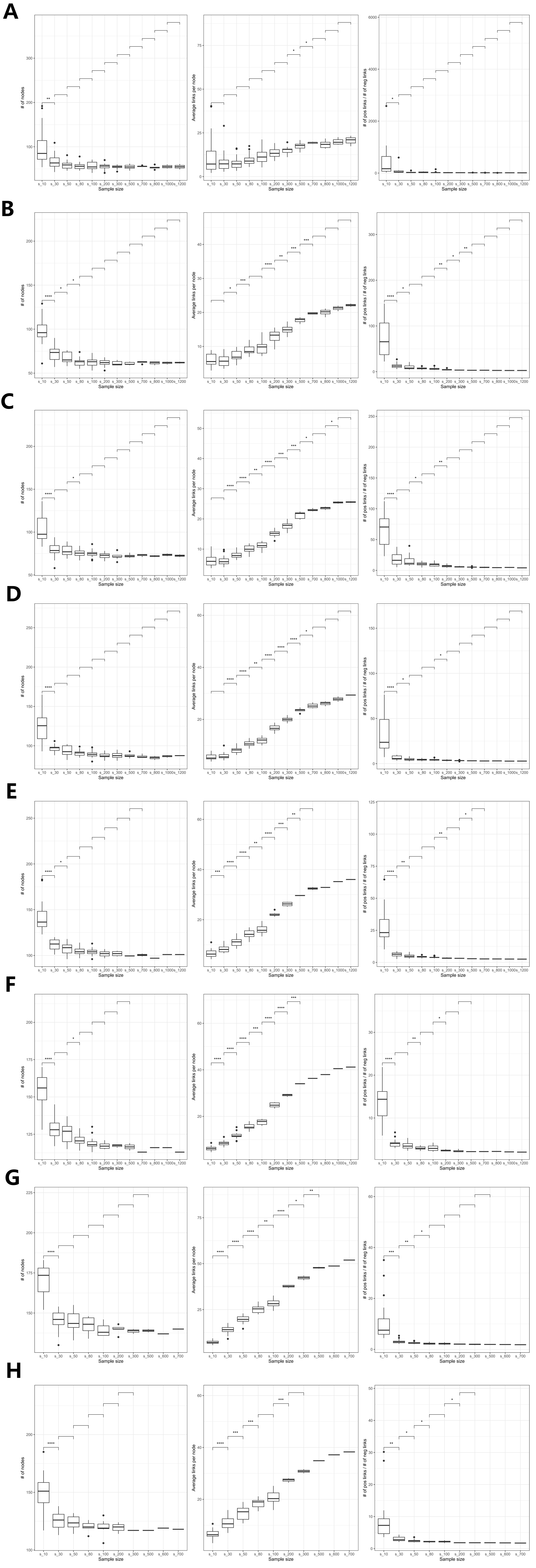

Supplementary Figure 3. The change of network node numbers, average links and positive-to-negative link ratios on different sample sizes for Spearman correlation networks. A, 0~1m. B, 1~3m. C, 3~6m. D, 6~12m. E, 12~18m. F, 18~24m. G, 24~36m. H, 36m+.

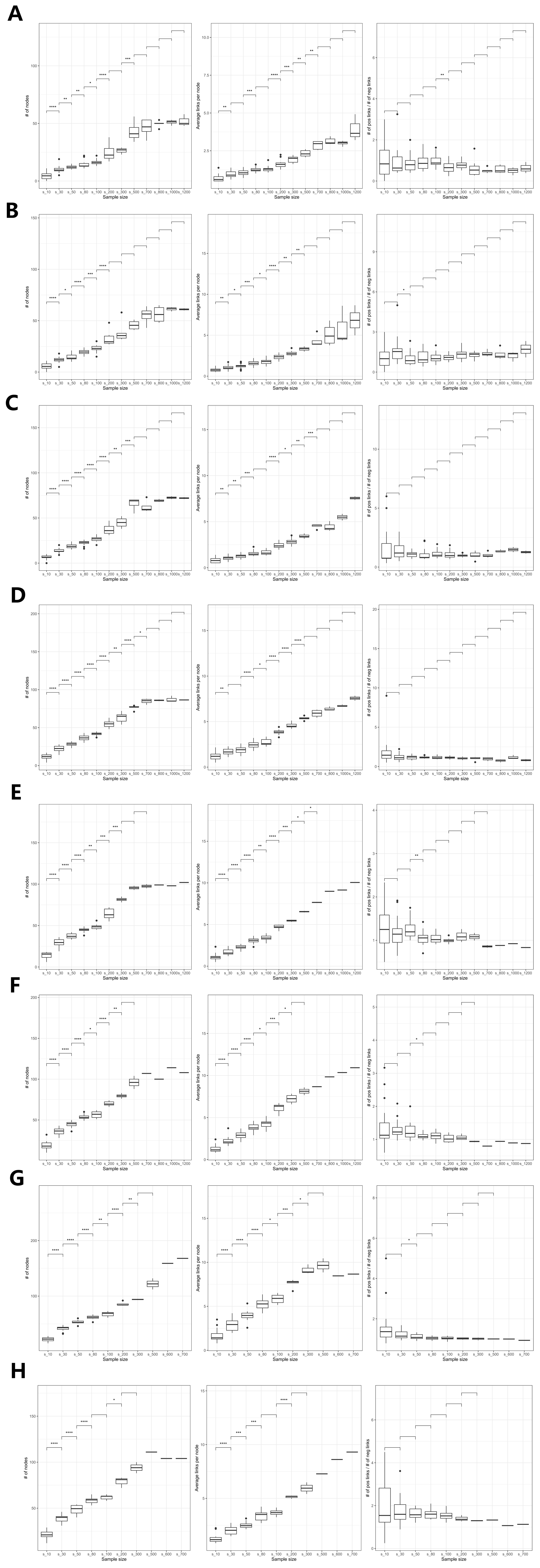

Supplementary Figure 4. The change of network node numbers, average links and positive-to-negative link ratios on different sample sizes for SparCC networks. A, 0~1m. B, 1~3m. C, 3~6m. D, 6~12m. E, 12~18m. F, 18~24m. G, 24~36m. H, 36m+.

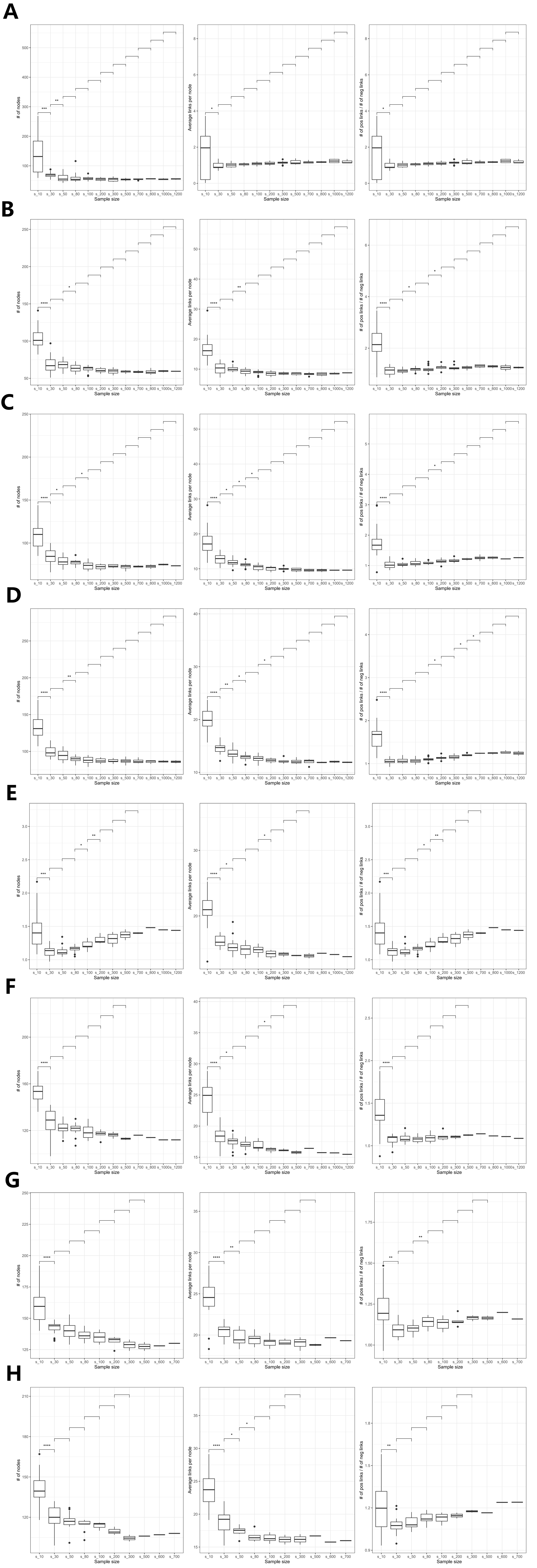

Supplementary Figure 5. The change of network node numbers, average links and positive-to-negative link ratios on different sample sizes for PBCDM-based networks. A, 0~1m. B, 1~3m. C, 3~6m. D, 6~12m. E, 12~18m. F, 18~24m. G, 24~36m. H, 36m+.

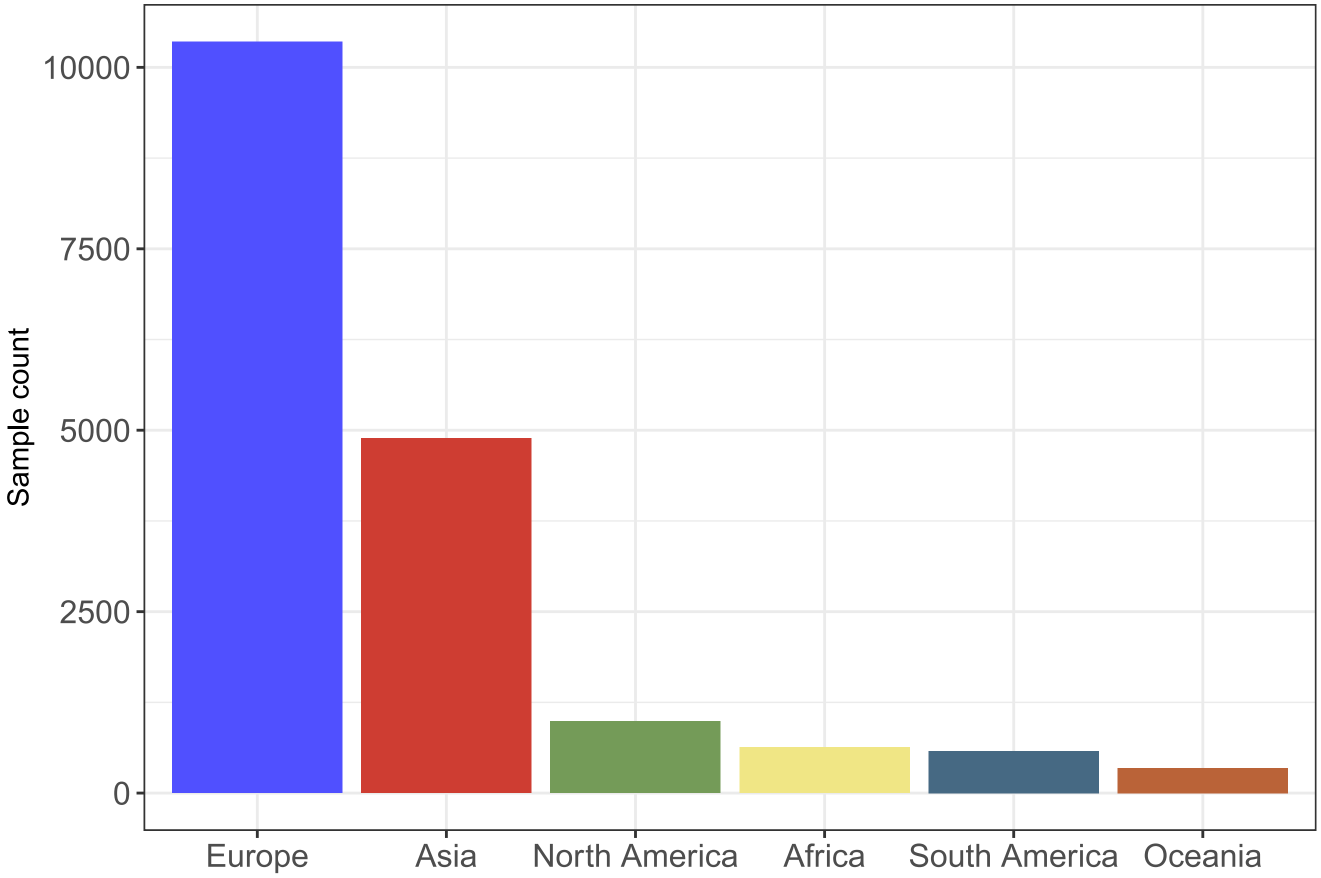

Supplementary Figure 6. Counts of samples from different continets. Different color bars indicate varied continents.

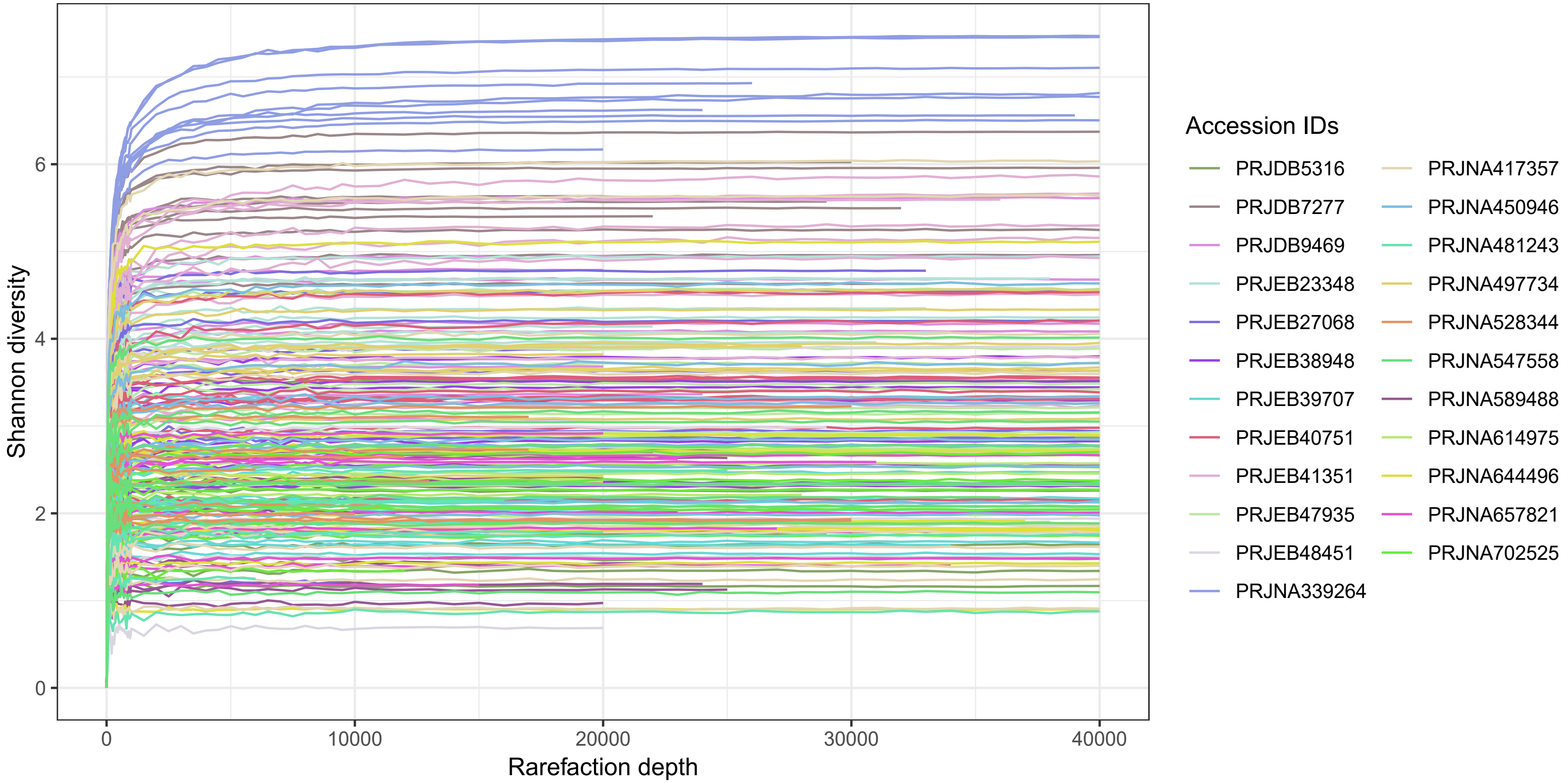

Supplementary Figure 7. Rarefaction test on shannon diversity of 10 random selected samples from each public accessions. Different line colors indicate varied public datasets.

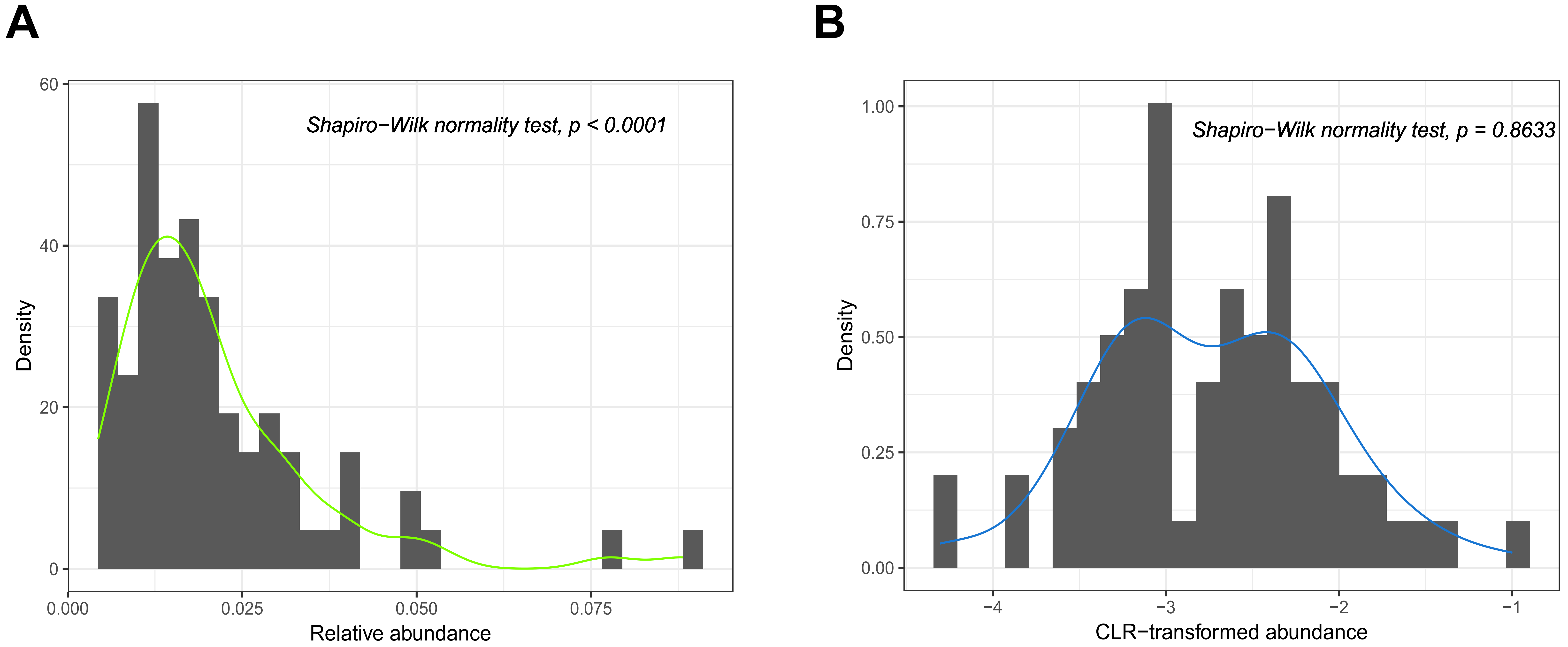

Supplementary Figure 8. Abundance distribution of genus *corynebacterium* before and after centered log-ratio (CLR) transformation. A, before transformation. B, after transformation. The green line indicates the curve fitting of the histogram of original relative abundance. The blue line indicates the curve fitting of the histogram of CLR-transformed abundance.

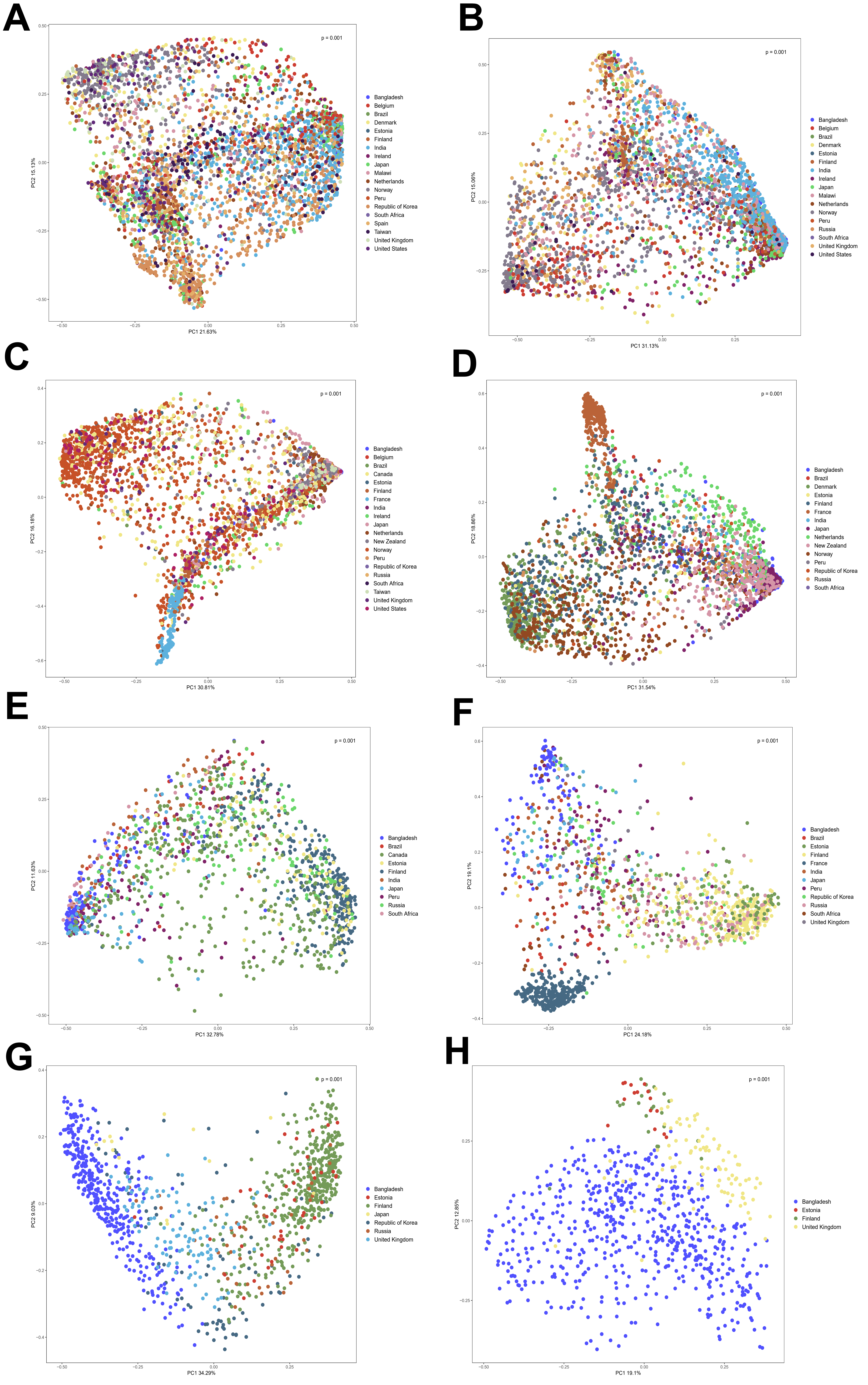

Supplementary Figure 9. The principal coordinate analysis of pooled samples on sample sourced countries. A, 0~1m. B, 1~3m. C. 3~6m. D, 6~12m. E, 12~18m. F, 18~24m. G, 24~36m. H, 36m+. Dots in different colors indicate samples from varied countries.

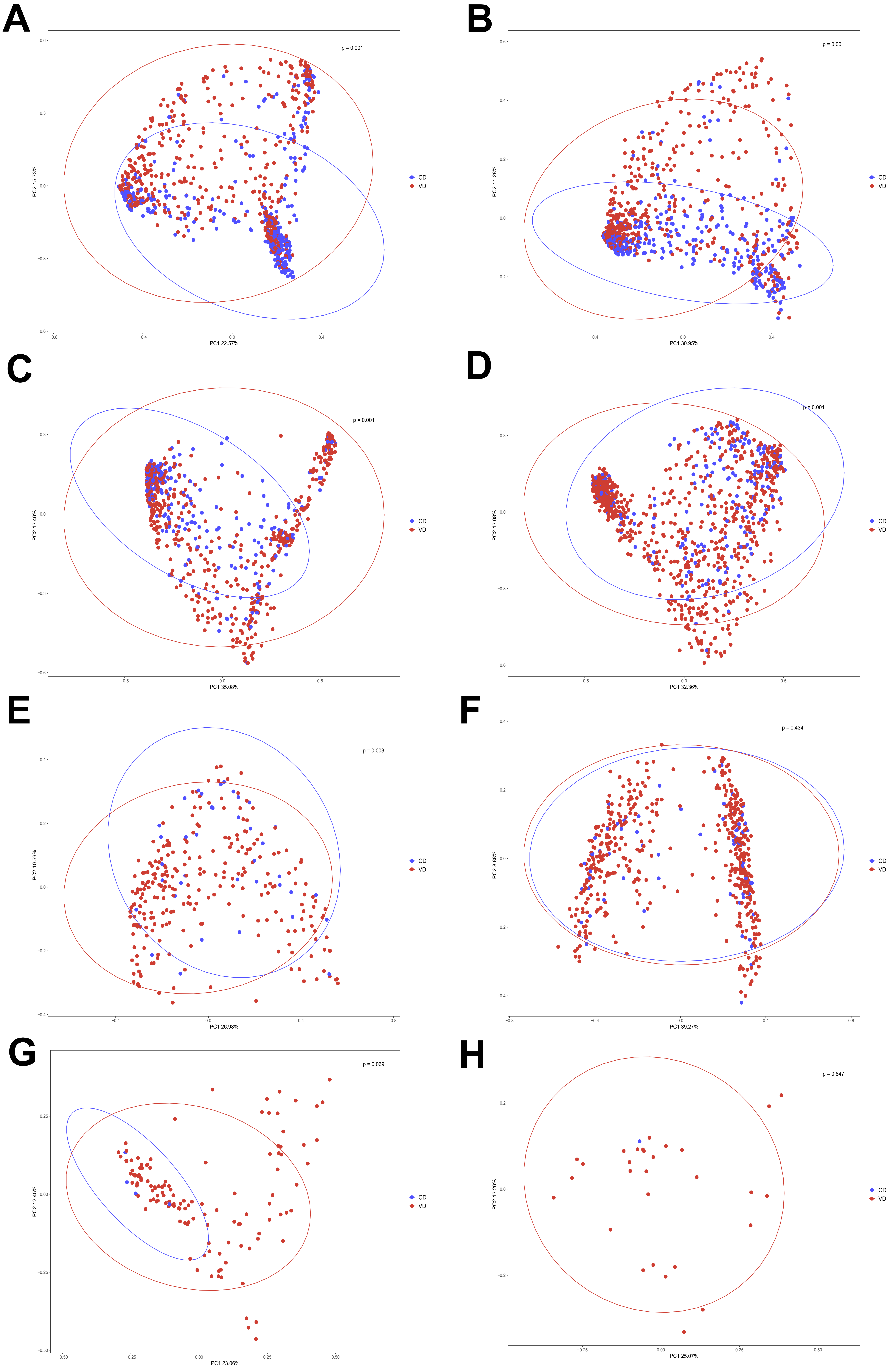

Supplementary Figure 10. The principal coordinate analysis of pooled samples on delivery modes. A, 0~1m. B, 1~3m. C, 3~6m. D, 6~12m. E, 12~18m. F, 18~24m. G, 24~36m. H, 36m+. CD, C-section. VD, virginal delivery. These groups are indicated by colored circles showing the 95% confidence intervals.

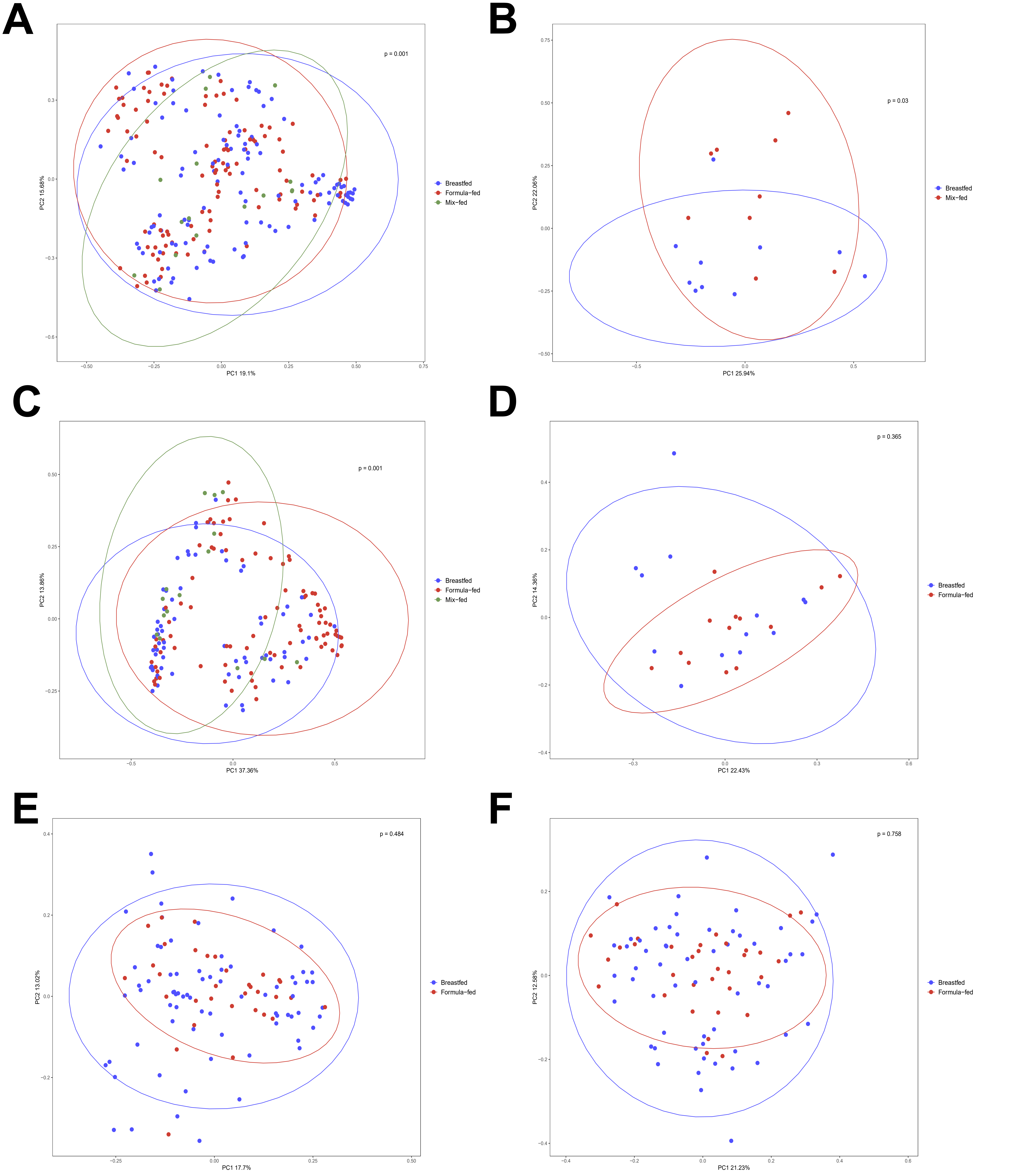

Supplementary Figure 11. The principal coordinate analysis of pooled samples on breast-feeding patterns. A, 0~1m. B, 1~3m. C, 3~6m. D, 18~24m. E, 24~36m. F, 36m+. No relevant breastfeeding information is available for samples in 6~12m and 12~18m age groups. These groups are indicated by colored circles showing the 95% confidence intervals.

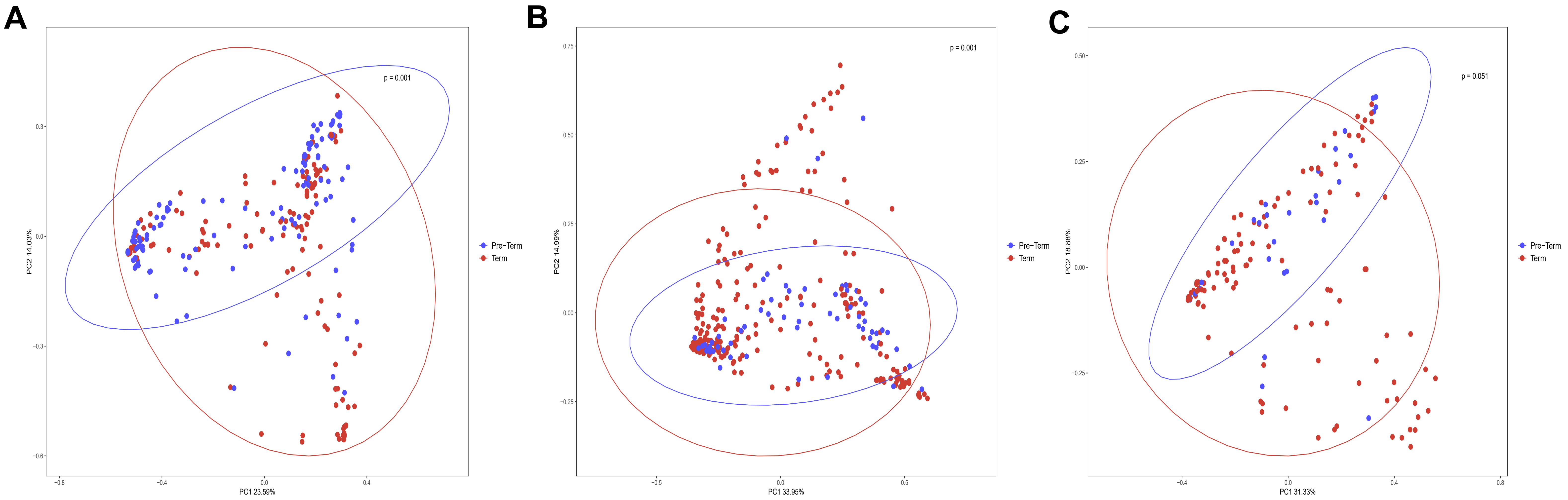

Supplementary Figure 12. The principal coordinate analysis of pooled samples on term mode, specifically full term and pre-term. A, 0~1m. B, 1~3m. C, 3~6m. No relevant term information for samples aged over 6 months. These groups are indicated by colored circles showing the 95% confidence intervals.
