# Supplementary material for: A novel robust network construction and analysis workflow for mining infant microbiota relationships
Source: mSystems. 2024 Dec 31;10(2):e01570-24. doi: 10.1128/msystems.01570-24 (PMC11834438; doi:10.1128/msystems.01570-24)
Supplement: Supplemental Table Captions — Captions for Tables S1 and S2. [file msystems.01570-24-s0004.docx]

Supplementary Table 1. Public amplicon sequencing datasets from 23 studies on infant microbiota.

Supplementary Table 2. Key genera detected at the core co-exist networks across varying age ranges.
